# Identification of crucial circRNAs in skeletal muscle during chicken embryonic development

**DOI:** 10.1186/s12864-022-08588-4

**Published:** 2022-04-28

**Authors:** Pengfei Wu, Kaizhi Zhou, Jin Zhang, Xuanze Ling, Xinchao Zhang, Li Zhang, Peifeng Li, Qingyu Wei, Tao Zhang, Xinglong Wang, Genxi Zhang

**Affiliations:** 1 College of Animal Science and Technology, Yangzhou University, Yangzhou, 225009 China; 2 College of Animal Science, Shanxi Agricultural University, Taiyuan, 030032 China

**Keywords:** circRNA, Embryo, Skeletal muscle, Growth and development, ceRNA

## Abstract

**Background:**

Chicken provides humans with a large amount of animal protein every year, in which skeletal muscle plays a leading role. The embryonic skeletal muscle development determines the number of muscle fibers and will affect the muscle production of chickens. CircRNAs are involved in a variety of important biological processes, including muscle development. However, studies on circRNAs in the chicken embryo muscle development are still lacking.

**Results:**

In the study, we collected chicken leg muscles at 14 and 20-day embryo ages both in the fast- and slow-growing groups for RNA-seq. We identified 245 and 440 differentially expressed (DE) circRNAs in the comparison group F14vsF20 and S14vsS20 respectively. GO enrichment analysis for the host genes of DE circRNAs showed that biological process (BP) terms in the top 20 related to growth in F14vsF20 were found such as positive regulation of transcription involved in G1/S phase of mitotic cell cycle, multicellular organismal macromolecule metabolic process, and multicellular organismal metabolic process. In group S14vsS20, we also found some BP terms associated with growth in the top 20 including actomyosin structure organization, actin cytoskeleton organization and myofibril assembly. A total of 7 significantly enriched pathways were obtained, containing Adherens junction and Tight junction. Further analysis of those pathways found three crucial host genes *MYH9*, *YBX3, IGF1R* in both fast- and slow-growing groups, three important host genes *CTNNA3, AFDN* and *CREBBP* only in the fast-growing group, and six host genes *FGFR2, ACTN2, COL1A2, CDC42, DOCK1* and *MYL3* only in the slow-growing group. In addition, circRNA-miRNA network also revealed some key regulation pairs such as *novel_circ_0007646-miR-1625-5p, novel_circ_0007646-miR-1680-5p, novel_circ_0008913-miR-148b-5p, novel_circ_0008906-miR-148b-5p* and *novel_circ_0001640-miR-1759-3p.*

**Conclusions:**

Comprehensive analysis of circRNAs and their targets would contribute to a better understanding of the molecular mechanisms in poultry skeletal muscle and it also plays an important guiding role in the next research.

**Supplementary Information:**

The online version contains supplementary material available at 10.1186/s12864-022-08588-4.

## Background

China is rich in local chicken genetic resources, most of which have the advantages of high meat quality and strong stress resistance. However, the growth rate of local chicken resources is usually slow, which seriously limits their industrialization. In recent years, the market share of high-quality broilers has been increasing. Therefore, breeding high-quality broilers with Chinese indigenous chicken is the direction. Skeletal muscle is crucial to the broiler industry and it can directly affect production performance [1, 2]. The study on the skeletal muscle mechanism of chickens would make contributions to future breeding work.

At present, a large number of studies have involved the mechanism of skeletal muscle growth and development of genes [3, 4]. In recent years, the diverse mechanisms and functions of non-coding RNAs (mainly including miRNA, lncRNA and circRNA) have attracted much attention. The function of miRNAs in skeletal muscle has been gradually revealed in chicken. Studies have shown that some specific miRNAs related to skeletal muscle development were labeled myomiRs including *miR-1, miR-133* and *miR-206* [5, 6]. With in-depth research, numbers of non-skeletal muscle-specific miRNAs have also been found to be closely related to skeletal muscle growth [7–9]. LncRNA widely exists in eukaryotic organisms and it could modulate transcription, epigenetic modifications, protein/RNA stability, translation, and posttranslational modifications by interacting with DNA, RNAs and/or proteins [10]. In the study of chicken skeletal muscle, it was found that lncRNA-six1 could activate the gene Six1 in cis-acting by encoding a micro peptide of about 7.26 kDa and promoting the proliferation and differentiation of chicken myoblasts [11]. In addition, *lncRNA-six1* was also found to affect the proliferation and differentiation of myoblasts as a ceRNA by adsorbing *miR-1611* [12].

CircRNA, as a class of non-coding RNAs without 5′ caps and 3′ tails, has also been widely studied in recent years. CircRNA was covalently closed RNA molecules generated by back splicing of mRNA [13] and it could participate in various biological processes through a variety of mechanisms [14]. Shen et al. [15] identified an abundant circular RNA *circTMTC1*, which was expressed significantly higher in layers than in broilers at E10, E13 and E16. Furthermore, they found it could inhibit chicken skeletal muscle satellite cells (SMSCs) differentiation by sponging *miR-128-3p*. Yin et al. [16] identified a novel circular RNA *circFAM188B*, which encodes a novel protein *circFAM188B*-103aa to promote proliferation and inhibit differentiation in chicken SMSCs. Chen et al. [17] found that *circHIPK3* could act as a sponge of *miR-30a-3p* and exert a counteractive effect of *miR-30a-3p* by promoting the proliferation and differentiation of myoblasts.

At present, studies on circRNA regulating skeletal muscle development of chicken at embryonic stages were still limited. Age of 14-day embryo is the key period for myoblasts in skeletal muscle to proliferate and differentiate into myotubes and finally fuse into muscle fibers, which have been basically fixed at 20-day embryo age. We collected leg muscles of 14 and 20-day embryos of Bian chickens for RNA-seq and expected to identify the key circRNAs of skeletal muscle growth. This work would provide guidance for the next functional research.

## Results

### Quality control of raw data

The RNA extracted in the experiment is of high quality and fully meets the needs for sequencing (Table S1). The quality control results of raw data were shown in Table S2. Clean bases of samples have 11.86G (S14_1) at least. The percentage of the clean base with Q20 and Q30 was more than 98.00% (F20_4) and 93.99% (F20_4) respectively. The GC content of the samples ranged from 45.26 to 48.49%. All the further analyses were based on clean data with high quality.

### Differential expression analysis

**Fig. 1:**
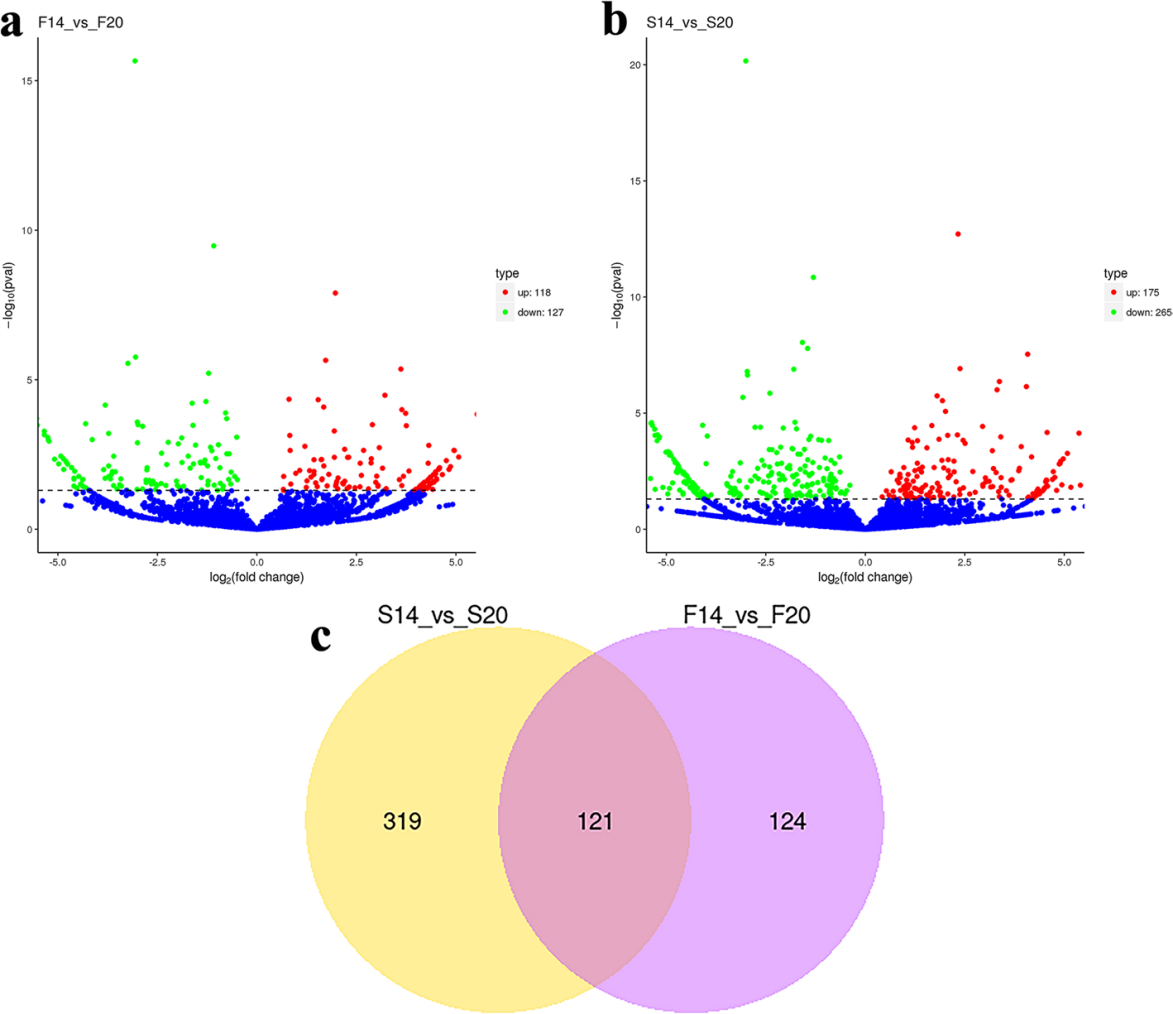
Differentially expressed (DE) circRNA analysis. **a** DE circRNAs for F14vsF20; **(b)** DE circRNAs for S14vsS20; **(c)** Venn diagram for DE circRNAs of the fast- and slow-growing groups

**Fig. 2:**
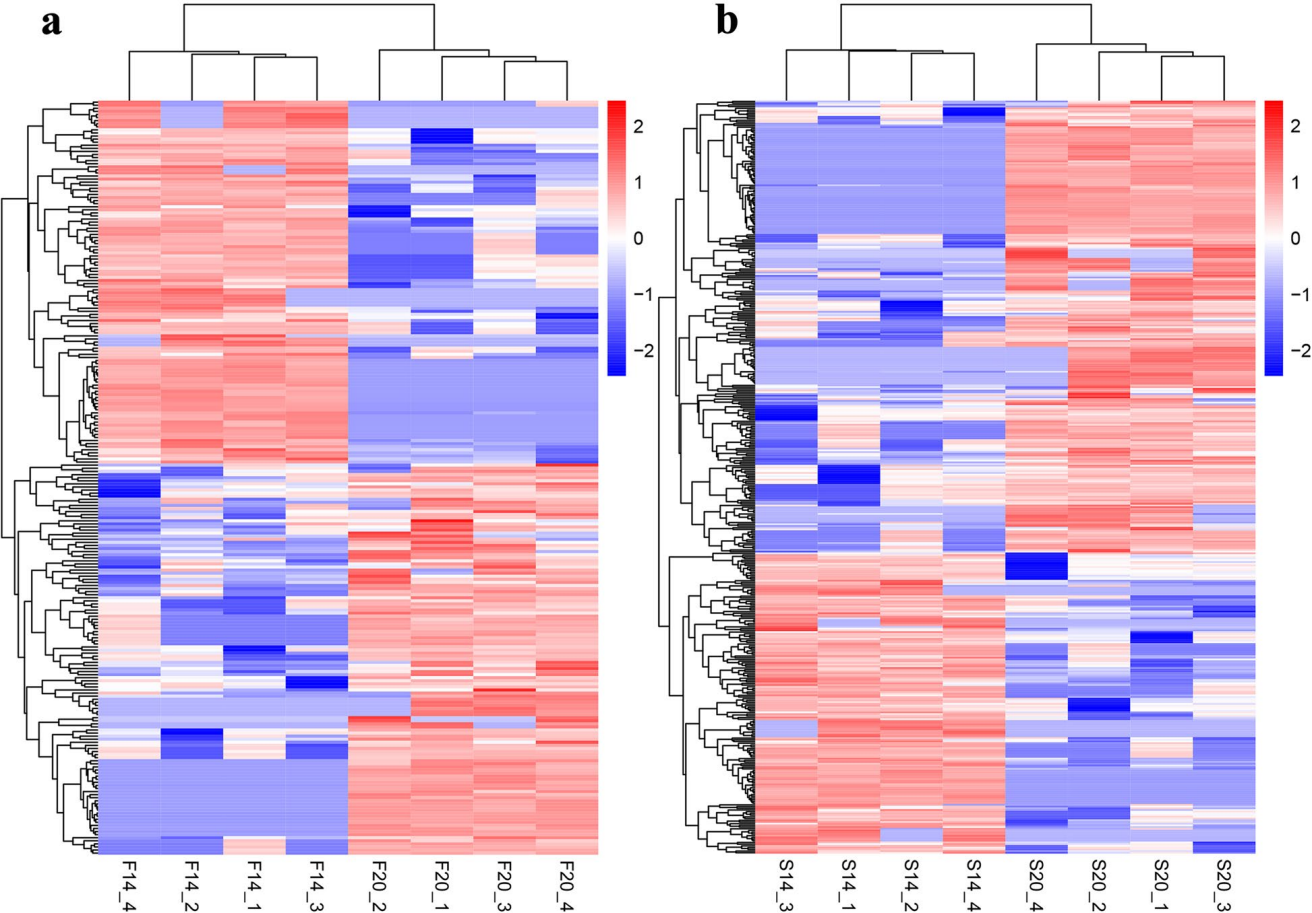
Results of hierarchical clustering analysis. **a** Hierarchical clustering results for differentially expressed (DE) circRNAs in F14vsF20; **(b)** Hierarchical clustering results for DE circRNAs in S14vsS20

Differential expression analysis showed that 245 differentially expressed (DE) circRNAs were obtained in F14vsF20 with *P*-value ≤0.05. Compared with 20-day embryo ages, there are 118 up-regulated and 127 down-regulated circRNAs in 14-day embryo (Fig. 1a). In the comparison group S14vsS20, we identified 440 DE circRNAs, including 175 up-regulated and 265-down regulated DE circRNAs (Fig. 1b). Further analysis showed that there were 121 co-differentially expressed circRNAs in the two comparison groups F14vsF20 and S14vsS20 (Fig. 1c). In addition, a heatmap of DE circRNAs in differentially comparison groups based on transcript per million (TPM) values is shown in Fig. 2. Samples in the same group are clustered together, which shows that the repeatability of samples within the group is reliable.

### Functional analysis for host genes of differentially expressed circRNAs

Because most circRNAs are derived from middle exons of protein-coding genes, the processing of circRNAs can affect the splicing of their precursor transcripts, leading to altered gene expression outcomes [18]. GO and KEGG pathway enrichment for the host genes of DE circRNAs was performed in F14vsF20 and S14vsS20 respectively.

**Fig. 3:**
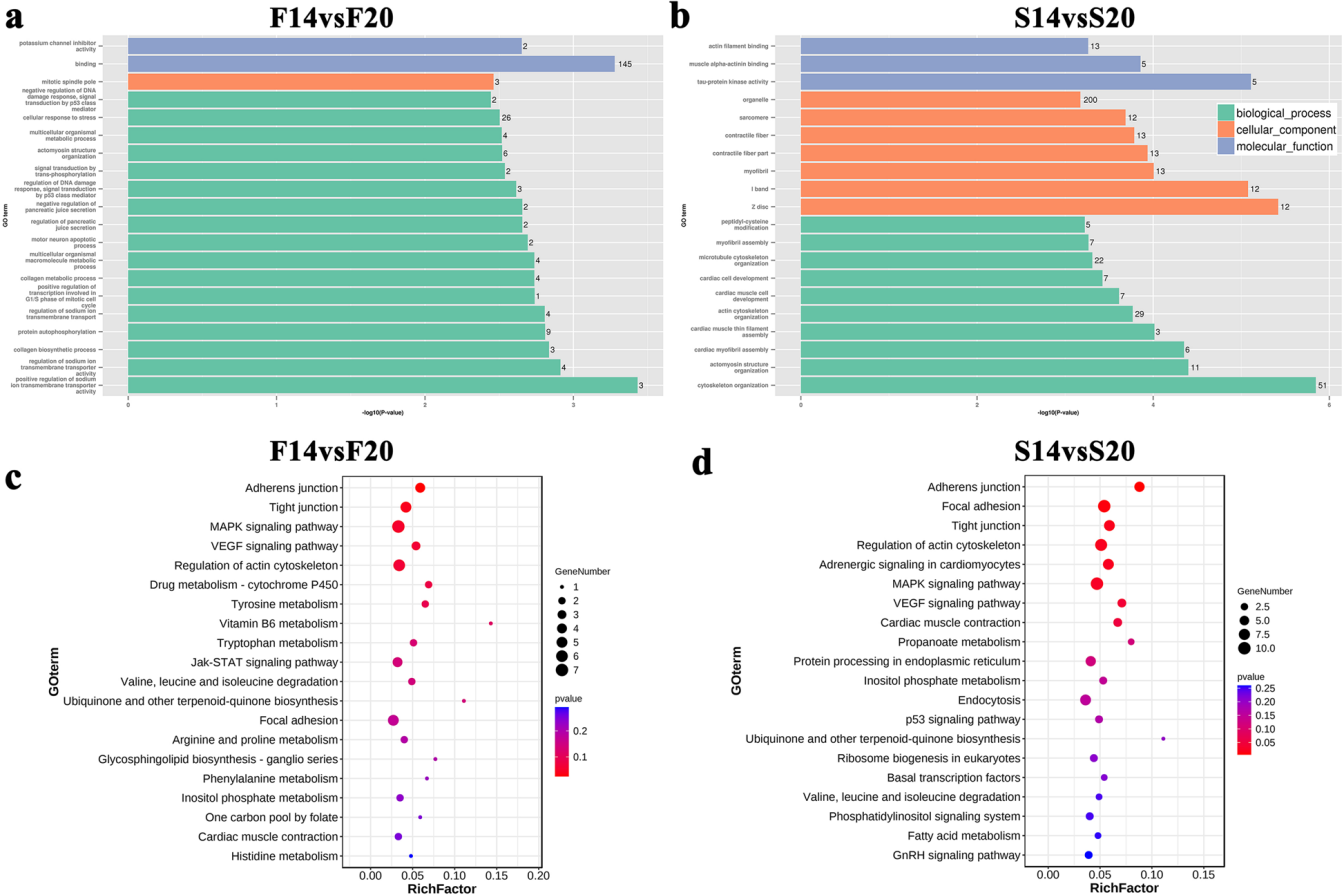
Functional enrichment analysis for host genes of the differentially expressed (DE) circRNA. **a** GO enrichment analysis of F14vsF20; **(b)** GO enrichment analysis of S14vsS20; **(c)** KEGG pathway enrichment of F14vsF20; **(d)** KEGG pathway enrichment of S14vsS20

In group F14vsF20, there are 17 biological process (BP) entries in the top 20 GO terms, some of which are related to growth and development (Fig. 3a), including positive regulation of transcription involved in G1/S phase of mitotic cell cycle, multicellular organismal macromolecule metabolic process and multicellular organismal metabolic process. In another comparison group, we found that there were 10 BP entries in the first 20 GO terms, and most of them were related to growth and development (Fig. 3b), containing actomyosin structure organization, actin cytoskeleton organization and myofibril assembly.

**Fig. 4:**
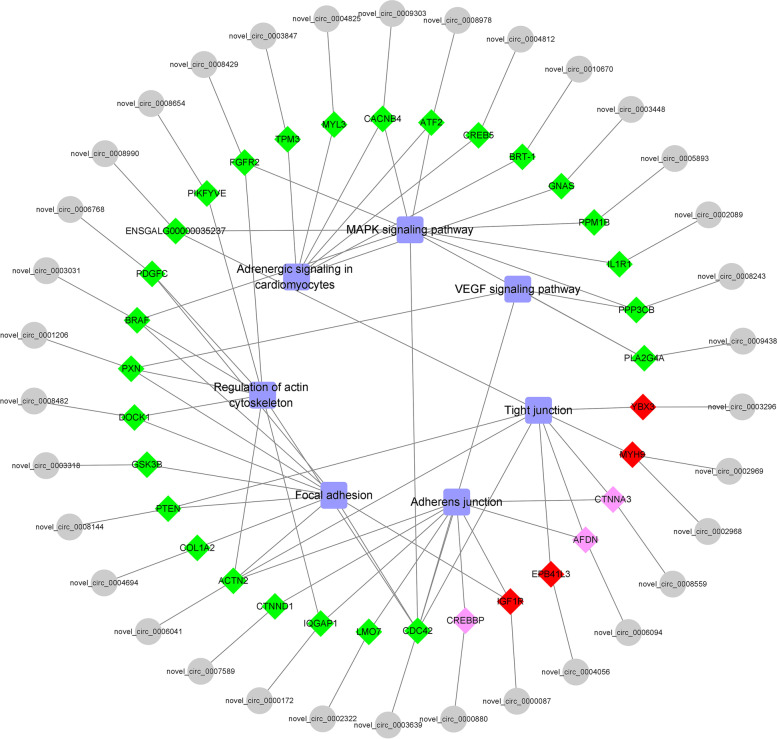
Visualization for the significant enrichment pathways and enriched genes of F14vsF20 and S14vsS20. NOTE: The innermost squares with blue represent the seven significantly enriched pathways; The middle layer represents enriched host genes: red nodes were simultaneously enriched in the significant pathways of the two comparison groups, pink nodes were only significantly enriched pathways of F14vsF20 group and green nodes were significantly enriched in S14vS20; The outermost gray circles represent the host circRNAs

The top 20 pathways of KEGG enrichment analysis are shown in Fig. 3c and d. In the F14vsF20 group, we found two significantly enriched pathways, Adherens junction and Tight junction, and they were closely related to growth. In addition, pathways MAPK signaling pathway and focal adhesion in the top 20 are also important to muscle development. Among the top 20 pathways in S14vsS20 group, we found seven significantly enriched pathways, including adherens junction and tight junction, which were the same as that in group F14vsF20. We selected all significant pathways and their enriched genes for visualization with Cytoscape. The result (Fig. 4) showed that four genes (red nodes) were simultaneously enriched in the significant pathways of the two comparison groups, and three genes (pink nodes) were only significantly enriched pathways of F14vsF20 group and all other genes (green nodes) were significantly enriched in S14vS20.

### Result of validation for DE circRNAs

**Fig. 5:**
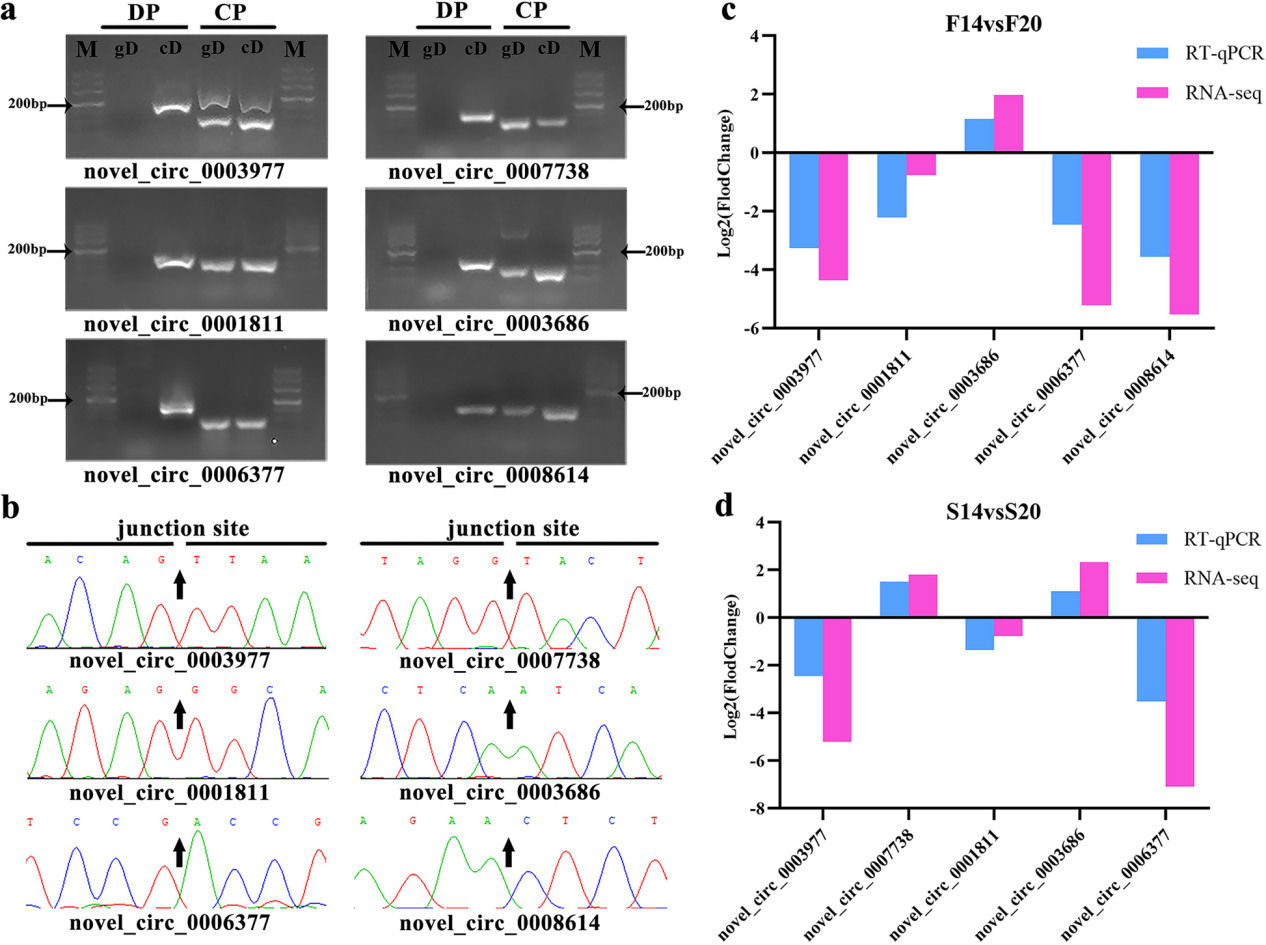
Validation of differentially expressed circRNAs. **a** agarose gel electrophoresis test for PCR products of divergent primers (DP) and convergent primers (CP) using cDNA (cD) and gDNA (gD). **b** Sanger sequencing confirmed the back-splicing junction of circRNAs; **(c)** RT-qPCR validation of five differentially expressed circRNAs in F14vsF20 and S14vsS20 respectively. NOTE: Original gels are presented in Supplementary Fig. S1; M: maker; DP: divergent primers; CP: convergent primers

A total of six DE circRNAs were selected for validation, and agarose gel electrophoresis showed (Fig. 5a) that all the convergent primers (CP) amplified bands both in the genomic DNA (gDNA) and cDNA, and the product sizes were also same. However, divergent primers (DP) only amplified product bands in cDNA. Sanger sequencing (Fig. 5b) for back-spliced junction (BSJ) sites was the same as RNA-seq sequence. Finally, result of RT-qPCR (Fig. 5c and d) for the 6 DE circRNAs were all consistent with the trends of RNA-seq. The all above results suggested that the sequencing results of cicRNA are reliable.

### CircRNA-miRNA networks

**Fig. 6:**
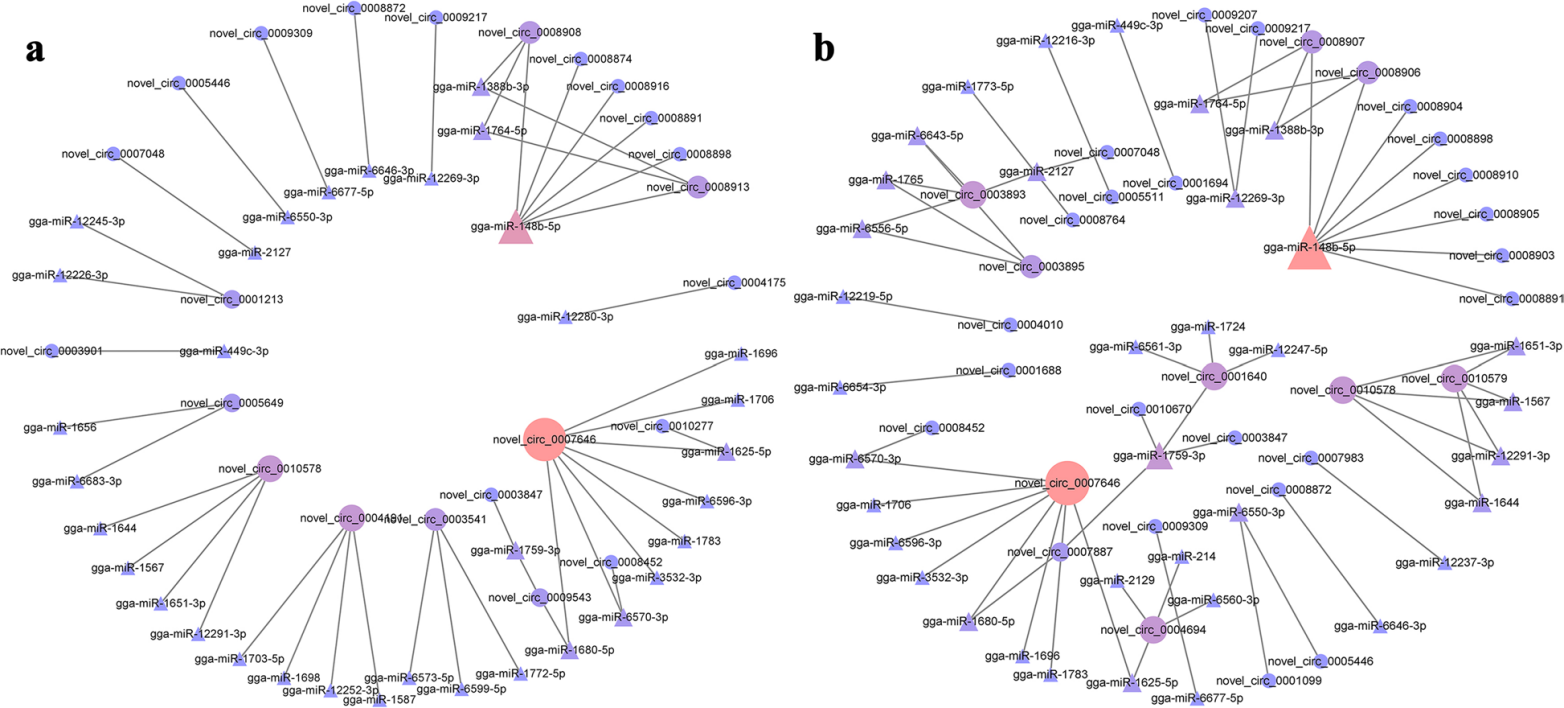
circRNA-miRNA interaction network. **a** circRNA-miRNA pairs of F14vsF20. **b** circRNA-miRNA pairs of S14vsS20. NOTE: Circles represent circRNAs and triangles represent miRNAs

The main mechanism of circRNAs may act as a miRNA sponge to modulate post-transcriptional regulation [19, 20]. We performed KEGG and GO functional enrichment analysis on miRNAs targeted by circRNA. Some pathways related to amino acid metabolism in the top 20 (Table S3 and S4) are enriched, including Cysteine and methionine metabolism, Arginine and proline metabolism, Biosynthesis of amino acids and Tyrosine metabolism. There are also some pathways associated with mRNA, such as RNA transport, RNA polymerase and mRNA surveillance pathway in the top 20. In addition, we also found Glycolysis / Gluconeogenesis and Fructose and mannose metabolism pathways related to carbohydrate metabolism. In the GO enrichment results, we found 55 biological process (BP) terms related to skeletal muscle both in F14vsF20 and S14vsS20 groups (Table S5 and S6). Based on the genes enriched in these entries, we constructed the DE cirRNA-miRNA relationship pairs for the two comparison groups and found 9453 and 16,635 pairs respectively. We showed cirRNA-miRNA pairs with energy≤ − 50 in Table S7 and S8. The lower the energy value, the more reliable the targeted binding relationship is. Finally we used them for visualization with the software Cytoscape (Fig. 6).

The NetworkAnalyzer plug-in in Cytoscape software was used to calculate the connection degree. CircRNAs or miRNAs with higher connection degree were identified as highly connected. *Novel_circ_0007646* had the highest degree both in the two groups, and *novel_circ_0007646-miR-1625-5p, −miR-1680-5p and -miR-6570-3p* pairs were also important in the two groups. The miRNA with the highest connectivity in the two groups was *miR-148b-5p. MiR-148b-5p-novel_circ_0008913* and -*novel_circ_0008908* pairs may play an important role in the fast-growing group, while *miR-148b-5p-novel_circ_0008906* and *-novel_circ_0008907* were significant in the slow-growing group. In addition, we also speculated that *miR-1759-3p- novel_circ_0001640* pairs may also significantly regulate the growth and development of skeletal muscle according to their degrees in the slow-growing group.

## Discussion

As a source of animal protein, chicken occupies an important share in the market of meat and it has provided the human diet across the globe with large amounts of protein [21]. Improving the quantity and quality of chicken meat has always been the goal of researchers in the past few decades. Many genes closely related to the development of skeletal muscle have also been revealed, such as *MSTN* [22], *MRFs* [23], *MEF2* [24], etc. In recent years, the research on non-coding RNA of skeletal muscle growth has gradually increased [8, 25, 26]. CircRNA, as one of the major non-coding RNAs, has rich and extremely important functions on a variety of biological processes, including skeletal muscle development [19, 27].

In the study, we collected chicken leg muscles of different embryonic ages for transcriptome sequencing and identified 245 and 440 DE circRNAs in F14vsF20 and S14vsS20. Slow-growing chickens presented more DE circRNAs than fast-growing chickens. It is speculated that different numbers of DE circRNAs in F14vsF20 and S14vsS20 may be caused by differences in the regulation of muscle development. GO enrichment for the host genes of DE circRNAs was performed in F14vsF20 and S14vsS20 respectively, and some BP terms related to skeletal muscle have been identified including myofibril assembly, actomyosin structure organization and protein autophosphorylation. KEGG enrichment analysis showed that two and seven pathways were significantly enriched in F14vsF20 and S14vsS20 respectively (Fig. 4) and most of them were closely related to skeletal muscle development. The KEGG pathway adrenergic signaling in cardiomyoocytes was enriched, which may be that the cardiac muscle and skeletal muscle are both striated muscles. Besides, the leg muscles contain a number of different tissues and blood vessels were also included in them. Hence, the VEGF signaling pathway was also significantly enriched. Figure 4 showed that red nodes, *MYH9, YBX3, IGF1R* and *EPB41L3* were simultaneously enriched in the significant pathways of the two comparison groups. Pink nodes, *CTNNA3, AFDN* and *CREBBP*, were only significantly enriched pathways of F14vsF20 group. All other genes (green nodes) were significantly enriched in pathways of S14vS20 and genes such as *FGFR2, ACTN2, COL1A2, CDC42, DOCK1* and *MYL3* are closely related to skeletal muscle growth.

The hierarchy of skeletal muscles runs from the top to bottom as muscles, fibre bundles, fibres, myofibrils and sarcomeres [28]. In cross-striated muscle, sarcomeres contained Myosin II (thick) filaments and actin (thin) filaments [29], which were both involved in the regulation of the contraction of striated muscle [30]. The myosin II subfamily is the largest class of myosins and includes skeletal, cardiac and smooth muscle myosins, as well as non-muscle myosin-2 (NM2) isoforms [31]. The class II myosin forms the filaments in muscle and non-muscle cells as a hexameric protein complex, consisting of two myosin heavy chain (*MyHC*) subunits, two regulatory light chains (RLCs) and two essential light chains (ELCs) [32]. Together, the ELCs and the RLCs are named myosin light chains (MLCs), which are important regulators of actin-myosin interactions. Vertebrates hold three paralog genes (*MYH9, MYH10* and *MYH14*), which are located in different chromosomes and encode three NMHC2 isoforms (*NMHC2A, 2B* and *2C*, respectively) [31]. Before muscle-specific myosin II (MM II) is organized in mature myofibrils to carry out that role, however, non-muscle isoforms of myosin II (NM II) are present in premyofibrils and nascent myofibrils that lead to mature myofibril formation [33]. During embryonic development, from 14 to 20 embryonic ages, we found that *MYH9* was found as the host gene of DE circRNAs in the significantly enriched pathways of both F14vsF20 and S14vsS20 (Fig. 5). The results suggest that *novel_circ_0002968* and *novel_circ_0002969*, and the host gene *MYH9* may play an important role in myofibril formation. In addition, *MYL3* as the host gene of DE *novel_circ_0004825*, was found to be significantly enriched in group S14vsS20 and it was a member of the myosin light chains (MLCs). Study [34] has found that *MYL3* could bind calcium ions, promote muscle development, and participate in the contraction of striated muscles.

*YBX3* is a member of Y-box protein family, which contains a conserved cold shock domain (CSD), enabling these proteins to bind to single-stranded nucleic acids [35]. DNA- and RNA-binding capabilities allow members of this family to perform diverse functions, including regulation of transcription, splicing, translation, and mRNA stability [36]. Studies have confirmed that the post-translational phosphorylation of *MSY3 (YBX3)* by Akt kinase could rescue down-regulation of myogenin caused by binding of *MSY3* in skeletal muscle [37, 38]. *IGF1R* is an *IGF-1* receptor with a transmembrane location that activates *PI3K/Akt* signaling and possesses tyrosine kinase activity, and its expression is significant in terms of myoblast proliferation and normal muscle mass maintenance [39]. *IGF1R*, as the host gene of *novel_circ_0000087*, was significantly enriched in adherens junction and focal adhesion pathways in both F14vsF20 and S14vsS20.

*CTNNA3, AFDN* and *CREBBP* were found to be significantly enriched in pathways of F14vsF20. The study of *CTNNA3* and *AFDN* in skeletal muscle has not been reported, but there are many studies on them in tumor cells, mostly related to tumor cell proliferation and migration [40, 41]. The host gene of DE *novel_circ_0008559* and *novel_circ_0006094* were *CTNNA3* and *AFDN*, respectively. We speculated that they may also regulate the growth of skeletal muscle cells. *CREB*-binding protein (*CREBBP*, or in short *CBP*) is a kind of lysine (K) acetyltransferases (*KAT*) belonging to the *KAT3* family of proteins known to modify histones, as well as non-histone proteins, thereby regulating chromatin accessibility and transcription [42]. Svensson et al. [43] revealed that *CREBBP* was required for the control and maintenance of contractile function and transcriptional homeostasis in skeletal muscle of adult mice.

In comparison group S12vsS20, we also identified many host genes of DE circRNAs related to skeletal muscle development. Fibroblast Growth Factor Receptor 2 (*FGFR2*) was a member of *FGFR*s, which are a family of receptor tyrosine kinases expressed on the cell membrane that play crucial roles in both developmental and adult cells [44]. *FGFR2* has been proved to regulate the myogenesis of skeletal muscle [45, 46]. *ACTN2* was highly expressed in muscle where it acted as a major structural component of the contractile apparatus at the Z-line [47]. Sharma et al. [48] found that *col1a2*^*+*^ muscle progenitor cells contributed to new myofibers in normal muscle growth and also during muscle regeneration. Integrin/FAK pathway is required for C2C12 myoblast differentiation by regulating the expression of *MyoD* and *CDC42* [49]. *DOCK1* (also known as *Dock180*) is a prototypical member of a new family of atypical Rho GTPase activators and Laurin et al. [50] have identified *DOCK1* and *DOCK5* as critical regulators of the fusion step during primary myogenesis in mammals.

CircRNAs can serve as miRNA sponges to regulate the expression of mRNA by competitively adsorbing endogenous RNAs (ceRNAs). Ouyang et al. [51, 52] found that circSVIL expressed differentially among skeletal muscle at 11 embryo age (E11), 16 embryo age (E16), and 1 day post-hatch (P1). And experiment showed that *circSVIL* could promote myoblast proliferation and differentiation by sponging *miR-203* in chicken. Wei et al. [25] revealed that *circFNDC3AL* was differentially expressed between E10 and E19, E13 and E19 of ROSS 308 broilers and they further found *circFNDC3AL* could up-regulated *BCL9* expression to promote chicken skeletal muscle satellite cells proliferation and differentiation by binding to *miR-204*. KEGG and GO enrichment analyses were performed for miRNAs targeted by DE circRNAs as a sponge. Cysteine and methionine metabolism, RNA transport and Glycolysis / Gluconeogenesis were found in the top 20 pathways of the two groups. In the GO enrichment results, 55 biological process terms related to skeletal muscle were enriched including skeletal muscle cell differentiation, skeletal muscle satellite cell migration, skeletal muscle tissue regeneration, and so on. We selected the miRNAs in these items and constructed the miRNA-circRNA interaction network (Fig. 6).

CircRNAs generally have more than one miRNA binding site. For example, *ciRS-7* contains over 60 target sites for *miR-7* and can function as a *miR-7* sponge and influence miR-7 target gene expression [20]. In the study, *novel_circ_0007646* had the highest degree both in the two groups, and the targeted miRNAs include *miR-1625-5p*, *miR-1680-5p* and *miR-6570-3p* pairs, etc. In addition, one miRNA may also bind to multiple circRNAs. The targeted miRNA with the highest connectivity in the two groups was *miR-148b-5p*, and the predicted results showed that it could bind to circRNAs such as *novel_circ_0008913* and *novel_circ_0008908* in the fast-growing group, and *circ_0008906* and *novel_circ_0008907* in the slow-growing group. In the S14vsS20, we also found that the nodes in *miR-1759-3p-novel_circ_0001640* pairs both have a high degree, suggesting their important role in skeletal muscle development.

## Conclusion

In the study, we collected the leg muscles of 14 and 20 embryonic Bian chickens for circRNA-seq. We identified 245 and 440 DE circRNAs in the fast- and slow-growing groups, respectively. Functional enrichment analysis for the host genes of DE circRNAs revealed several important candidate genes, such as *MYH9, IGF1R, YBX3* and *CREBBP*. These host genes and their corresponding DE circRNAs may play a significant role in skeletal muscle development. In addition, the circRNA-miRNA network constructed based on ceRNA mechanism also found some crucial regulatory relationship pairs related to skeletal muscle, including *novel_circ_0007646-miR-1625-5p*, *novel_circ_0007646-miR-1680-5p*, *novel_circ_0008913-miR-148b-5p, circ_0008906- miR-148b-5p* and *novel_circ_0001640-miR-1759-3p*. These findings would further guide to carry out functional research of circRNA and it would also lay a foundation to further understand the mechanism of skeletal muscle development.

## Methods

### Animals and tissues

Bian chicken is an eminent native Chinese breed. Zhang et al. [53] established slow-growing and fast-growing groups with the gene-assisted selection for growth traits. Then the bidirectional selection of body weight at 16-week was further carried out for six generations. The 16-week body weight of female fast-growing and slow-growing Bian chickens in seventh generation was 1615 ± 176 g and 921 ± 93 g, respectively. At the age of 300 days, twelve female and one male Bian chickens in seventh generation closing to the average weight were selected from the slow-growing and fast-growing Bian chicken groups. After artificial insemination, the half-sib fertilized eggs were collected respectively in the two groups. They were incubated with a temperature of 37 °C and humidity of 60% until 14 or 20-day embryo ages (14E and 20E). The eggshell was removed and the chick embryos were decapitated rapidly. At the same time, a small amount of allantoic fluid at 14E and blood at 20E were collected for sex identification. Then the left leg muscles of female chicken embryos were collected and frozen in liquid nitrogen immediately. Body weight at 300 days of female/male chicken for parents and the body weight of offspring at 14/20-day embryo was shown in Table S10.

We dissected the chicken embryos and preliminarily determined their gender by gonadal observation because hens had regressed right gonad. And then the *CHD1* gene was amplified using allantoic fluid or the full blood to further identify the sex of chicken embryos. The agarose gel electrophoresis results of female embryos would show two bands, while male embryos showed one band. Finally, the left leg muscles of female Bian chicken embryos were used for RNA-seq and four biological replicates were set in this experiment.

### RNA quantification and qualification

Total RNA was extracted with TRIzol reagent. RNA degradation and contamination were monitored on 1% agarose gels. RNA purity was checked using the NanoPhotometer® spectrophotometer (IMPLEN, CA, USA). RNA integrity was assessed using the RNA Nano 6000 Assay Kit of the Bioanalyzer 2100 system (Agilent Technologies, CA, USA).

### The construction of cDNA library and sequencing

The ribosomal RNA was depleted from total RNA using the rRNA Removal Kit following manufacturer′s instruction. First strand cDNA was reverse-transcribed using fragmented RNA and dNTPs (dATP, dTTP, dCTP and dGTP). RNA was degraded using RNase H, and second strand cDNA was synthesised using DNA polymerase I and dNTPs (dATP, dUTP, dCTP and dGTP). Remaining overhangs of double-strand cDNA were converted into blunt ends via exonuclease/ polymerase activities. After adenylation of 3′ends of DNA fragments, sequencing adaptors were ligated to the cDNA. The library fragments were purified (AMPure XP system) and library quality was assessed on the Agilent Bioanalyzer 2100 system. Finally, the qualified cDNA libraries were sequenced on the Illumina platform (NovaSeq 6000) and 150 bp paired-end reads were generated.

### Data analysis

Clean data (clean reads) were obtained by removing reads containing adapter, reads on containing ploy-N and low-quality reads from raw data. At the same time, Q20, Q30 and GC content of the clean data were calculated. All the downstream analyses were based on the clean data with high quality. Clean reads for each sample were mapped to the reference genome GRCg6a with the software Bowtie2 [54].

The circRNA were detected and identified using find_circ [19] and CIRI2 [55]. The raw counts were normalized using TPM [56]. DESeq2 [57] was used for differential expression analysis. Finally, circRNAs with *P*-value ≤0.05 were assigned as differentially expressed.

Gene Ontology (GO) enrichment analysis for host genes of differentially expressed (DE) circRNAs were implemented by the GOseq R package, in which gene length bias was corrected. GO terms with P-value≤0.05 were considered significantly enriched. KEGG is a database resource for understanding high-level functions and utilities of the biological system [58]. We used KOBAS software to test the statistical enrichment of differential expression genes or circRNA host genes in KEGG pathways.

### Validation for DE circRNAs

Six DE circRNAs were selected randomly for validation of RNA-seq in the two comparison groups F14vsF20 and S14vsS20, containing five same DE circRNAs. Divergent primers (DP) and convergent primers (CP) were designed with Primer 5.0 to amplify circRNAs back-spliced junction (BSJ) sites and linear mRNAs and they were shown in Table S9.

PCR products of divergent and convergent primers for cDNA and genomic DNA (gDNA) were analyzed by agarose gel electrophoresis. BSJ sites of circRNAs were further validated by sanger sequencing at Sango Biotech Co. Ltd. (Shanghai, China). RT-qPCR were performed on the platform QuantStudio 3 (Applied Biosystems, USA) with reagent Taq Pro Universal SYBR qPCR Master Mix (Q712, Vazyme Biotech Co., Ltd., China) according to the instruction. β-actin were selected as housekeeping gene and the 2^-△△CT^ method was used to calculate the relative expression of DE circRNAs.

### CircRNA-miRNA interaction analysis

CircRNAs as ceRNA can recruit miRNAs to regulate target gene expression [20], which was also one of the main regulation modes for circRNA. MiRanda software was used to predict the miRNA binding sites of DE circRNA, and 1200 and 1229 targeted miRNAs were obtained for DE circRNA of F14vsF20 and S14vsS20, respectively.

GO and KEGG enrichment analysis of miRNAs were performed using the Novomagic, a free online platform for data analysis (https://magic.novogene.com). Finally, we selected miRNAs enriched in GO terms related to “skeletal muscle” and constructed circRNA-miRNA interaction network.

## Supplementary Information

**Additional file 1.**

## Data Availability

The raw data of the study has been uploaded to the Sequence Read Archive (SRA) database and the accession number is PRJNA773377 (https://www.ncbi.nlm.nih.gov/bioproject/?term=PRJNA773377).

## Abbreviations

DE circRNA: Differentially expressed circRNA
14E: 14-day embryo
20E: 20-day embryo
F14/F20: 14/20-day embryo of the fast-growing group
S14/S20: S14/20-day embryo of the slow-growing group
GO: Gene Ontology
BP: Biological process
CC: Cell component
MF: Molecular function
KEGG: Kyoto Encyclopedia of Genes and Genomes
TPM: Transcripts Per Million
ceRNA: Competing endogenous RNA
gDNA: Genomic DNA
cDNA: Complementary DNA
SMSCs: Skeletal muscle satellite cells (SMSCs)
RT-qPCR: Real Time Quantitative PCR
DP: Divergent primers
CP: Convergent primers
