# Supplementary material for: Identification of crucial circRNAs in skeletal muscle during chicken embryonic development
Source: BMC Genomics. 2022 Apr 28;23:330. doi: 10.1186/s12864-022-08588-4 (PMC9052468; doi:10.1186/s12864-022-08588-4)
Supplement: Supplementary file 1 — Additional file 1. [file 12864_2022_8588_MOESM1_ESM.zip › Supplementary Material/Figure S1 Original images of agarose gel for Fig5. a..docx]

M

M

CP

DP

DP

CP


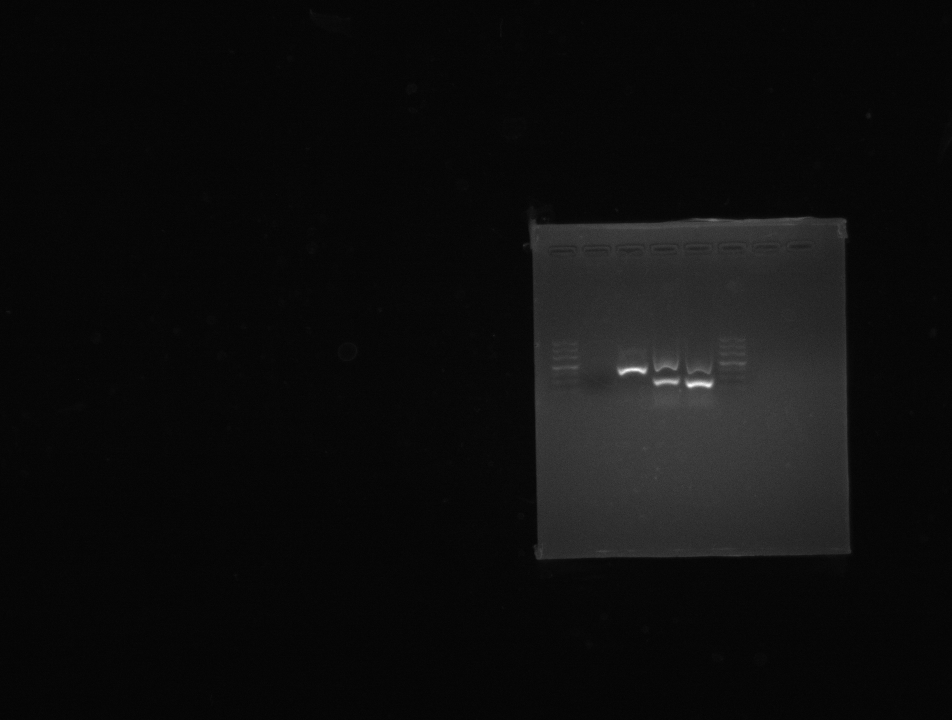

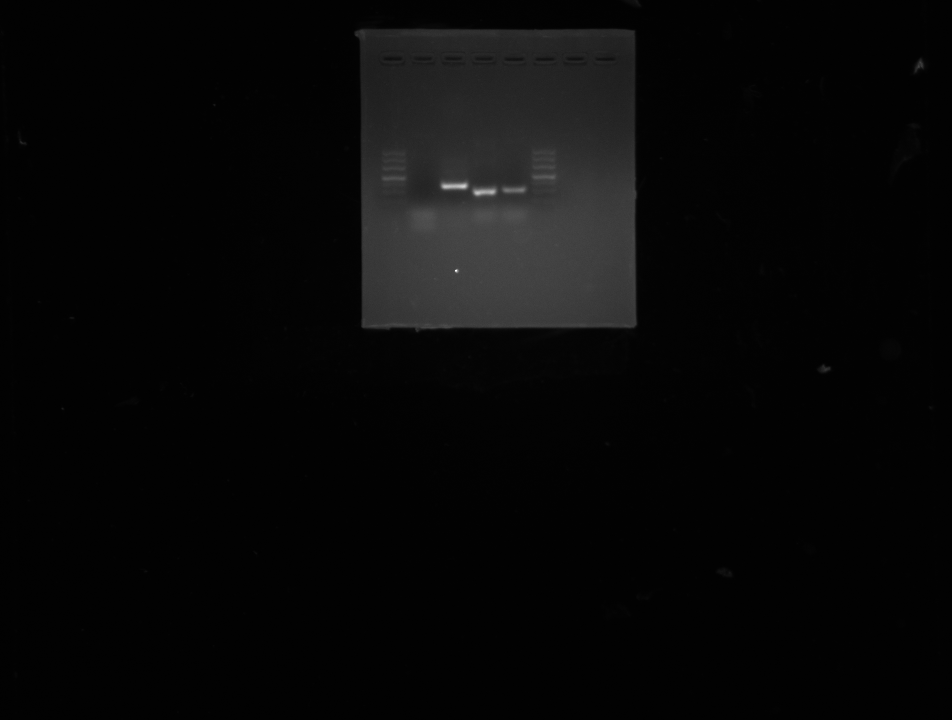


gD

gD

cD

cD

cD

gD

cD

gD

200bp

200bp

M

M

1. circRNA: novel_circ_0003977 b. circRNA: novel_circ_0007738


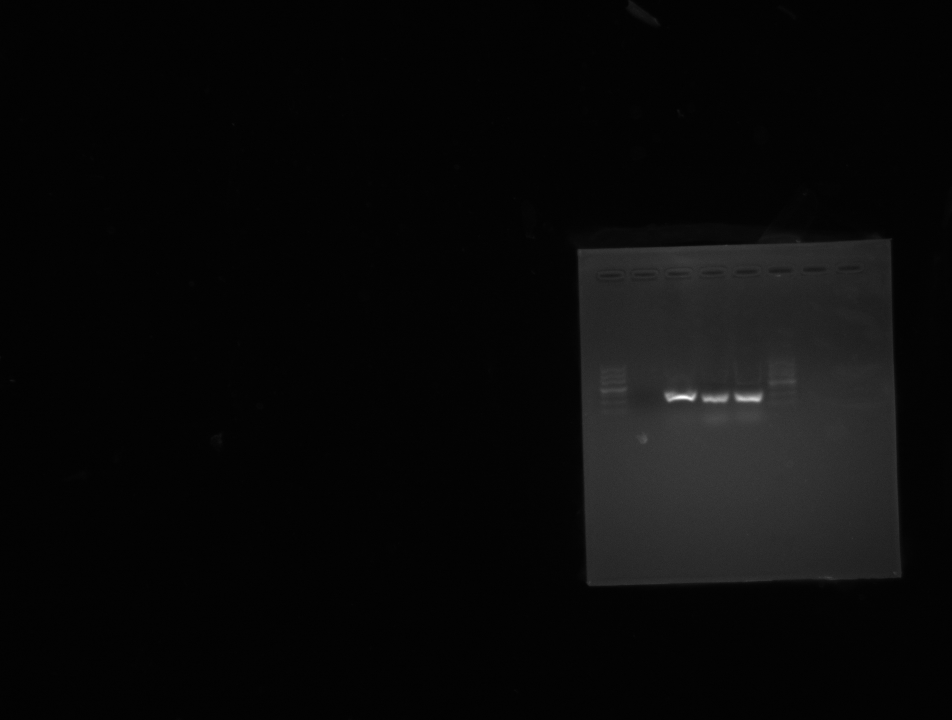

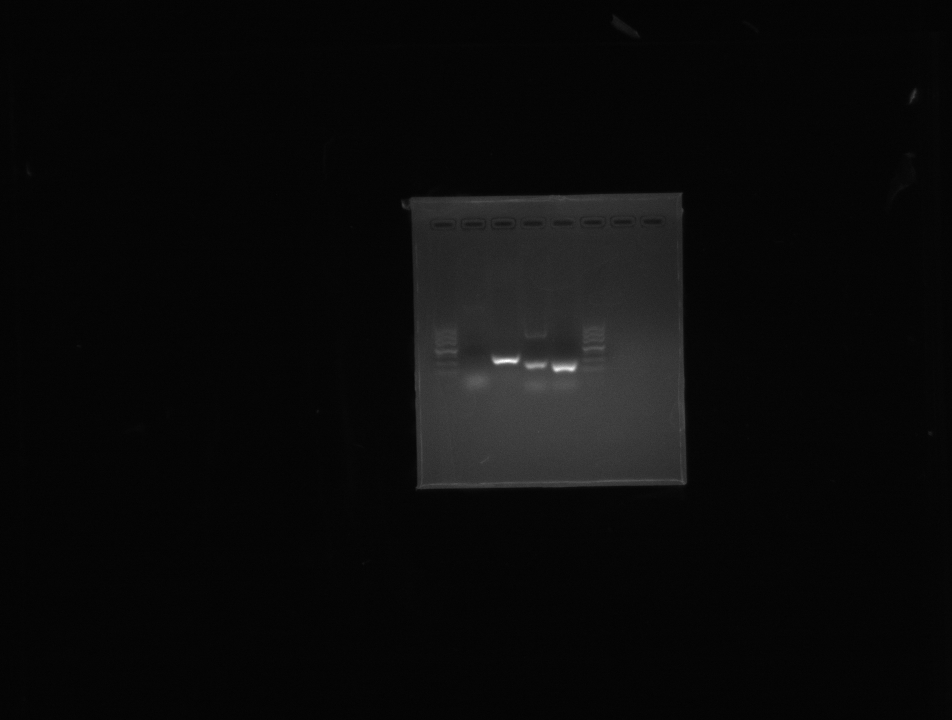


1. circRNA: novel_circ_0001811 d. circRNA: novel_circ_0003686


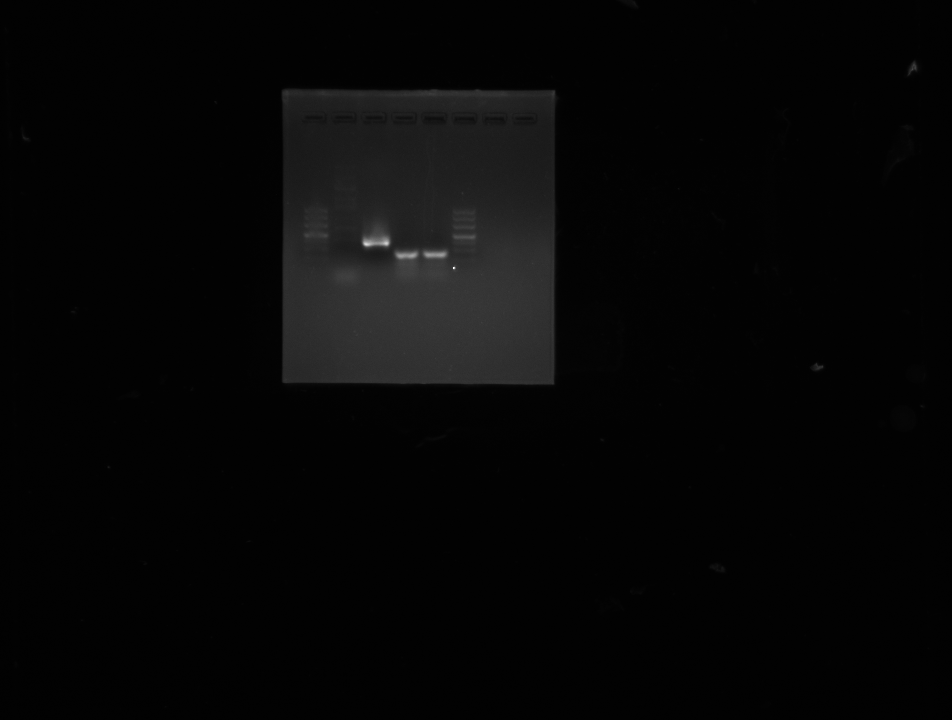

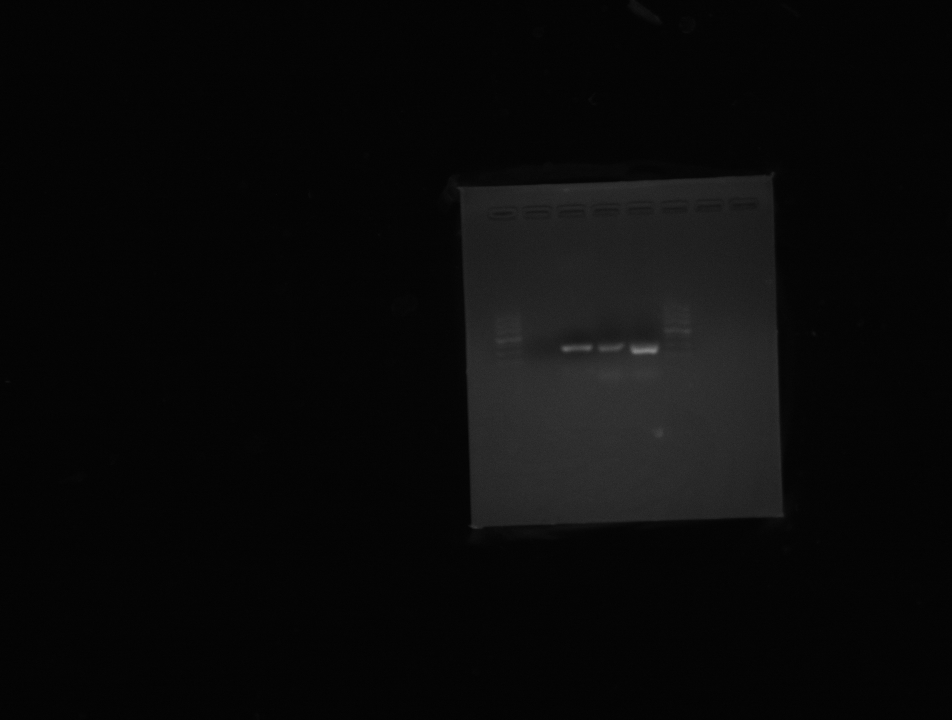


1. circRNA: novel_circ_0006377 f. circRNA: novel_circ_0008614

**Figure S1** Original images of agarose gel for Fig5. a. **NOTE:** M, maker; DP, divergent primers; CP, convergent primers; gD, genomic DNA (gDNA); cD, complementary DNA (cDNA).
