# Supplementary material for: Identification of crucial circRNAs in skeletal muscle during chicken embryonic development
Source: BMC Genomics. 2022 Apr 28;23:330. doi: 10.1186/s12864-022-08588-4 (PMC9052468; doi:10.1186/s12864-022-08588-4)
Supplement: Supplementary file 1 — Additional file 1. [file 12864_2022_8588_MOESM1_ESM.zip › Supplementary Material/Table S10 Statistics of body weight for fast- and slow-growing Bian chicken.docx]

**Table S10** **Statistics of body weight for fast- and slow-growing Bian chicken**

| Slow-growing Bian chicken | | Fast-growing Bian chicken | |
| --- | --- | --- | --- |
| The average body weight of female chicken at 300 days (g) | Body weight of male chicken at 300 days (g) | The average body weight of female chicken at 300 days (g) | Body weight of male chicken at 300 days (g) |
| 1252±24.73 | 1518 | 2248±63.11 | 3005 |
| Average body weight at 14-day embryo ages (g) | Average body weight at 20-day embryo ages (g) | Average body weight at 14-day embryo ages (g) | Average body weight at 20-day embryo ages (g) |
| 9.26±0.55^A^ | 32.60±1.70^B^ | 11.25±0.63^A^ | 39.26 ±2.40^B^ |

**NOTE:** ^A, B^ Different capital letters on one line in the same group indicate P ≤ 0.01.
